# The renal vasodilatation from β‐adrenergic activation in vivo in rats is not driven by K_V_7 and BK_Ca_ channels

**DOI:** 10.1113/EP091618

**Published:** 2024-03-09

**Authors:** Charlotte Mehlin Sorensen, Max Salomonsson, Anniek Frederike Lubberding, Niels‐Henrik Holstein‐Rathlou

**Affiliations:** ^1^ Department of Biomedical Sciences, Physiology of Circulation, Kidney and Lung University of Copenhagen Copenhagen Denmark; ^2^ Trelleborg Hospital Trelleborg Sweden; ^3^ Department of Biomedical Sciences, Physiology of Inflammation, Metabolism and Oxidation University of Copenhagen Copenhagen Denmark

**Keywords:** K^+^ channels, KCNQ, renal arteries, renal vascular resistance, sympathetic

## Abstract

The mechanisms behind renal vasodilatation elicited by stimulation of β‐adrenergic receptors are not clarified. As several classes of K channels are potentially activated, we tested the hypothesis that KV7 and BKCa channels contribute to the decreased renal vascular tone in vivo and in vitro. Changes in renal blood flow (RBF) during β‐adrenergic stimulation were measured in anaesthetized rats using an ultrasonic flow probe. The isometric tension of segmental arteries from normo‐ and hypertensive rats and segmental arteries from wild‐type mice and mice lacking functional K_V_7.1 channels was examined in a wire‐myograph. The β‐adrenergic agonist isoprenaline increased RBF significantly in vivo. Neither activation nor inhibition of K_V_7 and BK_Ca_ channels affected the β‐adrenergic RBF response. In segmental arteries from normo‐ and hypertensive rats, inhibition of K_V_7 channels significantly decreased the β‐adrenergic vasorelaxation. However, inhibiting BK_Ca_ channels was equally effective in reducing the β‐adrenergic vasorelaxation. The β‐adrenergic vasorelaxation was not different between segmental arteries from wild‐type mice and mice lacking K_V_7.1 channels. As opposed to rats, inhibition of K_V_7 channels did not affect the murine β‐adrenergic vasorelaxation. Although inhibition and activation of K_V_7 channels or BK_Ca_ channels significantly changed baseline RBF in vivo, none of the treatments affected β‐adrenergic vasodilatation. In isolated segmental arteries, however, inhibition of K_V_7 and BK_Ca_ channels significantly reduced the β‐adrenergic vasorelaxation, indicating that the regulation of RBF in vivo is driven by several actors in order to maintain an adequate RBF. Our data illustrates the challenge in extrapolating results from in vitro to in vivo conditions.

## Abstract (highlights)

- **What is the central question of this study?**
  Does activation of K_V_7 or BK_Ca_ channels drive renal β‐adrenergic vasodilatation?
- **What is the main finding and its importance?**
  K_V_7 and BK_Ca_ channels contribute significantly to renal β‐adrenergic vasorelaxation in vitro but have no effect in vivo although inhibition of K_V_7 and BK_Ca_ channels significantly reduce baseline renal blood flow. These findings highlight the importance of in vivo studies where several different mechanisms may compensate each other.

## 1 INTRODUCTION

The mechanism behind the renal vasodilatation induced by β‐adrenoceptor activation remains to be determined in vivo. Most focus has been on the α‐adrenergic receptors as they dominate in the renal vasculature. However, besides regulating the release of renin from the juxtaglomerular cells and the tubular Na^+^ reabsorption, β‐adrenergic receptors also regulate renal vascular resistance. As the kidney receives ∼20% of cardiac output, changes in renal vascular resistance can have a huge impact on arterial blood pressure. The tone of renal resistance vessels is mainly controlled by intracellular Ca^2+^ concentration ([Ca^2+^]_i_) in the vascular smooth muscle cells (VSMC) (Salomonsson et al., 2004). Changes in [Ca^2+^]_i_ are largely driven by influx of Ca^2+^ through L‐type voltage‐gated Ca^2+^ channels (Salomonsson & Arendshorst, 1999). Therefore, the membrane potential (*V*_m_) of VSMCs plays a central role in the regulation of renal vascular resistance. Potassium channels can greatly affect the resting membrane potential in VSMCs. In renal VSMCs, this potential has been measured to be approximately −55 to −40 mV (Chilton et al., 2011; Loutzenhiser et al., 1997), which is close to the activation threshold for L‐type Ca^2+^ channels (Brayden & Nelson, 1992; Buhrle et al., 1984). Thus, changes in the conductance of renal vascular K^+^ channels have the potential to change renal vascular resistance. At physiological K^+^ concentrations, the electrochemical driving force for K^+^ is directed outward. Opening of K^+^ channels leads to hyperpolarization of VSMCs and vasodilatation, whereas closure leads to depolarization and vasoconstriction.

Four major classes of K^+^ channels (calcium activated (K_Ca_), inward rectifier (Kir), voltage‐gated (Kv) and ATP sensitive (K_ATP_)) are present in the renal VSMCs (Salomonsson et al., 2017). The K_V_ channels are believed to buffer renal vasoconstriction as they are activated during depolarization. Two families of K_V_ channels are found in renal VSMCs, the K_V_1 and the K_V_7 families. The activation threshold for K_V_7 channels, encoded by the *KCNQ* gene family, is close to the resting membrane potential of renal VSMCs, −60 to −40 mV (Mackie & Byron, 2008). These channels could therefore participate in maintaining baseline renal vascular resistance as previously demonstrated in rats where general inhibition of K_V_7 channels significantly reduced renal blood flow (Salomonsson et al., 2015). K_V_7 channels are found in five subtypes, K_V_7.1–K_V_7.5. Of these, only K_V_7.4 has been shown to be expressed in the afferent arterioles (Salomonsson et al., 2015), whereas K_V_7.1, K_V_7.4 and K_V_7.5 have been found in the larger renal artery (Chadha, Zunke, Zhu et al., 2012). Large conductance calcium‐activated K^+^ channels (BK_Ca_) are also suggested to dampen vasoconstriction as they are activated by the increase in [Ca^2+^]_i_. However, they are also activated in response to many vasodilatory agonists working through cAMP (Salomonsson et al., 2017).

When renal vascular β‐adrenergic receptors are activated, adenylate cyclase is activated, increasing the intracellular levels of cAMP (Ruan et al., 1999) with subsequent activation of protein kinase A (PKA). Most of the K^+^ channels expressed in the renal vasculature are activated by PKA (Sorensen et al., 2012) including the K_V_7 family (van der Horst et al., 2020). K_V_7.1 channels have been shown to participate in the vasodilatation elicited by activation of the prostacyclin receptors (Baldwin et al., 2022). These receptors also activate PKA, similar to the downstream effect of β‐adrenergic receptor activation. Functional in vitro studies showed that K_V_7 channels, and in particular K_V_7.4 channels, are partly responsible for the β‐adrenergic vasodilatation in renal arteries from normotensive rats (Chadha, Zunke, Zhu et al., 2012). in vivo studies further showed that inhibition of K_V_7 channels increased renal vascular tone in anaesthetized normotensive rats (Salomonsson et al., 2015) indicating that these channels are active at baseline conditions and may be stimulated further upon activation of β‐adrenergic receptors. β‐Adrenergic receptors (β_1_ and β_2_) are expressed in the VSMCs in renal arteries (Boivin et al., 2001), and in hypertensive animal models the expression is increased (Michel et al., 1990). Stimulation of β‐adrenoceptors using, for example, isoprenaline elicits a significant renal vasodilatation in isolated kidneys (Martin & Broadley, 1995) and in anaesthetized normo‐ and hypertensive rats (Abdulla et al., 2009; El‐Gowilly et al., 2008). However, Chadha et al. showed, that β‐adrenergic renal vasorelaxation was absent in isolated renal arteries from hypertensive rats (SHR) (Chadha, Zunke, Zhu et al., 2012). This finding is intriguing as most studies report an increased sympathetic activity in hypertensive models, but also because renal blood flow has been shown to be reduced in young hypertensive rats (Dilley et al., 1984). Possibly, a reduced renal vasodilatation in response to activation of β‐adrenoceptors could play a role in the increased renal vascular resistance.

In the present study we aimed to elucidate whether opening of K_V_7 channels or BK_Ca_ channels contributes to β‐adrenergic renal vasodilatation in vivo, as previously found in vitro in normotensive rats. We further investigated if β‐adrenergic relaxation was present in the smaller segmental arteries isolated from hypertensive rats and whether closure of K_V_7 or BK_Ca_ channels affected this. Finally, we evaluated the functional role of K_V_7.1 channels in the renal vasculature utilizing isolated segmental arteries from wild‐type (WT) mice and mice with a knock‐in loss‐of‐function mutation in the K_V_7.1 channel (*KCNQ1* KI) as these channels have been shown to participate in the prostacyclin‐induced vasodilatation. In these experiments, we compared basal vascular tone and the effects of β‐ and α‐adrenergic stimulation.

Our hypotheses were that opening of K_V_7 or BK_Ca_ channels played a significant role in the renal β‐adrenergic relaxation both in vivo and in vitro and that this vasorelaxation would be present also in segmental arteries from hypertensive rats. Furthermore, we hypothesized that K_V_7.1 or BK_Ca_ played a significant role the renal vasorelaxation induced by activation of β‐adrenoceptors.

## 2 METHODS

### 2.1 Ethical approval

All procedures in this study were approved by the National Animal Experiments Inspectorate under the Ministry of Food, Agriculture and Fisheries of Denmark (licence numbers 2020‐15‐0201‐00547, 2023‐15‐0201‐01406 and breeding licence 2022‐15‐0202‐00156), by the Danish Working Environment Authority (GMO license 20230056376/4) and by the local veterinarian at University of Copenhagen (project number P20‐457). The experiments were ethically in accordance with the EU Directive 2010/63/EU for animal experiments. All animals were kept in a temperature‐controlled environment with a 12/12‐h light–dark cycle and had free access to standard chow (Altromin 1310, Lage, Germany) and tap water ad libitum. Trained animal caretakers were responsible for the rats’ well‐being until the experiments.

Experiments were performed in male Sprague–Dawley (SD) rats and male Spontaneously Hypertensive Rats (SHR) obtained from Taconic (Lille Skensved, Denmark). Further, male and female C57BL/6 mice with a loss‐of‐function mutation in *KCNQ1* and their WT littermates were used. These mice are described in Lubberding et al. (2021).

### 2.2 Mouse genotyping

DNA samples were lysed from mouse ear tissues by alkaline extraction. The DNA concentration was quantified by NanoDrop spectrophotometry (Thermo Fisher Scientific, Waltham, MA, USA), and then the DNA was used for PCR amplification using specific primers and HotStarTaq DNA polymerase, following the manufacturer's instructions. Genotyping was performed by digestion of the PCR amplification product using the restriction enzyme *Fau*I (New England Biolabs, Ipswich, MA, USA).

### 2.3 Isometric myograph recordings in rats

Isoflurane anaesthetized rats were killed by cervical dislocation. Kidneys were removed and placed in an ice‐cold dissection buffer with pH 7.4: NaCl 135 mM, KCl 5 mM, MgCl 1 mM, HEPES 10 mM, glucose 5 mM, CaCl_2_ 1 mM, albumin 5 g/l). Interlobar arteries with an inner diameter for SD rats of 380 ± 61 and for SHRs 352 ± 15 (*P* = 0.010) were isolated and cut to a length of 2 mm. Two stainless steel wires (Ø 40 µm) were inserted through the arterial lumen and the vessel was mounted in a wire myograph (Danish Myo Technology, Model 620M, Hinnerup, Denmark). The Physiological saline solution (PSS) solution in the chamber (NaCl 130 mM, KCl 4.7 mM, KH_2_PO_4_ 1.18 mM, MgSO_4_ 1.17 mM, NaHCO_3_ 14.9 mM, EDTA 0.026 mM, CaCl_2_ 1.6 mM, glucose 5.5 mM) was gassed with 5% CO_2_ and 95% O_2_ to maintain pH at 7.4. The chambers maintained a temperature of 37°C. (Lab‐Chart, ADInstruments, Oxford, UK) was used to record isometric tension.

#### 2.3.1 Normalization

All the experiments were performed at 90% *L*_100_ (Mulvany & Halpern, 1977), which is the stretch that generates a force in the vessel wall that corresponds to a transmural pressure of 100 mmHg. Vessel viability was tested by adding noradrenaline (NA; 10 µM) in a 60 mM K^+^ solution pH 7.4 (KPSS: NaCl 74.7 mM, KCl 60 mM, KH_2_PO_4_ 1.18 mM, MgSO_4_ 1.17 mM, NaHCO_3_ 14.9 mM, EDTA 0.026 mM, CaCl_2_ 1.6 mM and glucose 5.5 mM) twice at the start of the protocol and once at the end.

In segmental vessels from SD rats and SHRs, three different protocols were performed. The first tested if inhibition of voltage‐gated K_V_7 channels using XE‐991 would decrease β‐adrenergic vasodilatation (Iso + XE). Secondly, we tested if inhibition of the large conductance calcium‐activated K^+^ channel (BK_Ca_) using tetraethylammonium (TEA; Iso + TEA) would reduce β‐adrenergic vasodilatation. Lastly, the ability of K_V_7 channel inhibition using XE‐991 to reduce vasodilatation induced by forskolin‐stimulated activation of adenylate cyclase was examined (Forsk + XE).

#### 2.3.2 Experimental protocol for Iso + XE

Methoxamine was added (3 µM) to the chambers. When contraction was stable (after approx. 3 min) cumulative doses of isoprenaline (10 nM to 3 µM) were added every 3 min. After rinsing and resting (30 min), XE‐991 (10 µM) was added to the chamber. After 5 min methoxamine (3 µM) was added, and when the contraction was stable, the isoprenaline dose–response curve was repeated.

#### 2.3.3 Experimental protocol for Iso + TEA

The effect of inhibiting BK_Ca_ was tested using tetraethylammonium (TEA). At concentrations below 2 mM, TEA primarily inhibits BK_Ca_ (Orito et al., 1994; Su et al., 2001). Thirty minutes after the first isoprenaline dose–response curve (see above), TEA (1 mM) was added. After 5 min, the isoprenaline curve was repeated.

#### 2.3.4 Experimental protocol for Forsk + XE

Methoxamine was added (3 µM) to the chambers. When contraction was stable (after approx. 3 min) cumulative doses of forskolin (30 nM to 10 µM) were added every 3 min. After rinsing and resting (30 min), XE‐991 (10 µM) was added to the chamber. After 5 min methoxamine (3 µM) was added, and when the contraction was stable, the forskolin dose–response curve was repeated.

### 2.4 Isometric myograph recordings in mice

WT mice and mice with homozygous loss‐of‐function mutation in the K_V_7.1 channel (KI) were killed by cervical dislocation. Kidneys were removed and placed in an ice‐cold dissection buffer with pH 7.4. Interlobar arteries were isolated and cut to a length of 1–2 mm. Two stainless steel wires (Ø 40 µm) were inserted through the arterial lumen and the vessel was mounted in a wire myograph in heated PSS (37°C) gassed with 5% CO_2_ and 95% O_2_ to maintain pH at 7.4. The vessels were tested for viability as described above.

#### 2.4.1 Experimental protocol

Methoxamine was added in increasing doses (0.3, 1 and 3 µM) to the chambers. When contraction was stable (at 3 µM methoxamine after approx. 3 min) cumulative doses of isoprenaline (10 nM to 10 µM) were added every 3 min. After rinsing and resting (30 min), methoxamine (3 µM) was added to the chamber. The effect of the K_V_7.2–7.5 channel stimulator flupirtine was then evaluated by adding flupirtine in increasing doses (10 nM to 10 µM). Hereafter, the entire protocol was repeated using 5 min pretreatment with 10 µM XE‐991 before methoxamine dose–response, methoxamine + isoprenaline and methoxamine + flupirtine.

### 2.5 Surgical preparation for in vivo measurement of rat renal blood flow

Anaesthesia was induced by 5% isoflurane delivered in 65% nitrogen and 35% oxygen. The left carotid artery was catheterized for arterial blood pressure measurement using a Statham P23‐dB pressure transducer (Gould, Oxnard, CA, USA). Two catheters in the right jugular vein ensured infusion of saline (0.9%) and muscle relaxant (Nimbex; GlaxoSmithKline, 0.85 mg/ml, Brøndby, Denmark) at 20 µL/min. After tracheotomy, the rat was connected to a small animal ventilator (Ugo Basile, Gemonio, Italy; tidal volume: 8 ml/kg bw; frequency: 69 breaths/min) and placed on a heating table to maintain a constant body temperature (37°C). A final isoflurane concentration of 1%–2% maintained sufficient anaesthesia. The abdominal aorta and the left kidney were exposed through laparotomy. A PE‐10 catheter, with a tapered and bent tip, was introduced into the left iliac artery, carefully moved through the abdominal aorta and placed in the left renal artery to administer test agents directly into the renal artery in order to minimize systemic effects. A six‐port injection valve (IDEX Health & Science, Oak Harbor, WA, USA) was used for the administration of substances into the renal artery. The infusion rate was increased from 10 to 288 µL/min when substances were administered. Renal blood flow (RBF) was measured using a flow meter (Transonic T420; Ithaca, NY, USA) connected to an ultrasonic flow probe (1PRB) placed around the left renal artery. The left ureter was catheterized (PE‐10 connected to PE‐50) to ensure free urine flow. After surgery, 30 min recovery was allowed before experiments. After completion of the experiments the rats were killed by decapitation while still under anaesthesia.

### 2.6 Experimental protocol for in vivo measurement of rat renal blood flow

#### 2.6.1 Experimental protocol for Iso + XE

The β‐adrenergic agonist isoprenaline (0.1 µM) was infused intrarenally for 3 min. After stabilization of RBF (∼10 min), the K_V_7 channel inhibitor XE‐991 (5 µM) was infused and 4 min later isoprenaline (0.1 µM) was infused again. After 30 min washout a second baseline isoprenaline infusion was performed. Hereafter, 4 min pretreatment with K_V_7 channel activator flupirtine (10 µM) was initiated and the isoprenaline response was repeated.

#### 2.6.2 Experimental protocol for Iso + TEA

The baseline isoprenaline (0.1 µM) response was tested during a 3 min intrarenal infusion. After stabilization of RBF (∼10 min), the BK_Ca_ channel inhibitor TEA (1 mM) was infused and 4 min later isoprenaline (0.1 µM) was infused again. After 15 min washout a second baseline isoprenaline infusion was performed.

#### 2.6.3 Experimental protocol for Forsk + XE

The adenylate cyclase activator forskolin (0.5 µM) was infused intrarenally for 3 min. After ∼15 min, XE‐991 (5 µM) was infused and 4 min later the forskolin response was repeated. After 30 min washout, a second baseline forskolin infusion was performed. Hereafter, 4 min pretreatment with flupirtine (10 µM) was initiated and the forskolin response was repeated.

### 2.7 Drugs and chemicals

All chemicals were obtained from Sigma‐Aldrich, Søborg, Denmark. XE991 and flupirtine were dissolved in dimethyl sulfoxide (DMSO) and then further dissolved in PSS to final concentrations used in the myograph experiments. Methoxamine, isoprenaline and forskolin were dissolved in PSS.

In the in vivo experiments, drugs were dissolved to a concentration 10.4 times higher than the estimated plasma concentrations for renal artery infusion rate of 288 µL/min. Renal arterial plasma concentrations of the administered agents were the estimated plasma concentrations. Concentrations were calculated assuming a haematocrit of 40% (Probst et al., 2006) and an average renal plasma flow of 3 mL/min. In preliminary experiments, we found that the concentrations of DMSO used in this study did not affect RBF or vessel tension in the wire myograph. All substances in the in vivo experiments were further diluted in saline.

### 2.8 Statistics

For statistical analysis the SigmaStat (Systat Software, San Jose, CA, USA) software was used. Results are presented as means ± SD. Relative values in the myograph experiments are based on the methoxamine‐induced vasoconstriction. Delta values in the in vivo experiments are based on the baseline measurement (average of 1 min) immediately before infusing vasoactive drugs. A *P*‐value <0.05 was considered significant.

In the rat myograph experiments, four vessels from each rat were investigated simultaneously. In the mouse myograph experiments, two vessels from two different mice were investigated simultaneously. The genotype of the mice was unknown to the experimenter until after the final calculations. The number of experiments (*n*) refers to the number of animals used. The results from individual vessels from the same animal were averaged and used for further analysis.

Differences in BW, ID and vascular responses between groups were evaluated using Student's unpaired *t*‐test or one‐way ANOVA. Differences within groups were analysed using a two‐way ANOVA with repeated measures and Holm–Sidak as *post hoc* test. Changes in RBF were analysed using a Student's paired *t*‐test or repeated measures ANOVA followed by the Holm–Šidák *post hoc* test.

## 3 RESULTS

### 3.1 Isometric myograph recordings in rats

A total of 17 SD rats (BW 354 ± 44 g) and 15 SHRs (BW 330 ± 16 g, NS vs. SD; *P* = 0.067) were used for the myograph experiments.

#### 3.1.1 Iso + XE

**FIGURE 1:**
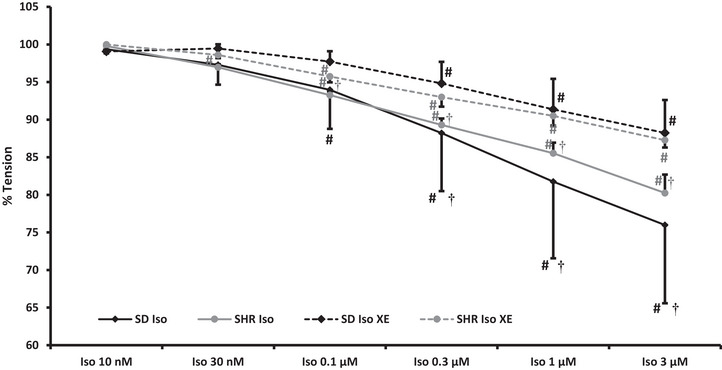
In isolated segmental arteries from normotensive (*n* = 8; black lines) and hypertensive (*n* = 6; grey lines) rats pre‐contracted with 3 µM methoxamine, isoprenaline elicits a significant dose‐dependent vasorelaxation (tension reduced to: SD rats: 76 ± 10% and SHRs: 80 ± 2%). Inhibition of K_V_7 channels using 10 µM XE‐991 reduced the β‐adrenergic renal vasorelaxation significantly in both rat strains (punctured lines; SD rats 88 ± 4% and SHRs 87 ± 1%). Data were compared using a 2‐way ANOVA with repeated measures. #*P *< 0.01 versus methoxamine; †*P *< 0.01 versus XE. Iso, isoprenaline; SD, Sprague–Dawley; SHR, spontaneously hypertensive rat; XE, XE‐991.

Segmental vessels from eight SD rats (32 vessels; inner diameter (ID): 392 ± 57 µm) and six SHRs (24 vessels; ID: 367 ± 54 µm; *P* = 0.140) were used in this protocol to examine whether the renal vasorelaxation elicited by β‐adrenoceptor activation could be reduced by closure of the K_V_7 channels. The methoxamine‐induced vasocontractions in SD rats (20.0 ± 4.7 mN/2 mm) and in SHRs (15.1 ± 2.3 mN/2 mm) were not different (nor after addition of XE‐991, *P* = 0.113 and *P* = 0.110). Adding isoprenaline in increasing doses induced a significant vasorelaxation in segmental vessels from both SD rats and SHRs (Figure 1) which were not different between strains. Inhibition of K_V_7 channels using XE‐991 induced a slight vasocontraction at baseline both in SD rats (1.9 ± 2.8 mN/2 mm, NS, *P* = 0.098) and in SHRs (0.9 ± 0.5 mN/2 mm, *P* = 0.011). XE‐991 significantly decreased the β‐adrenergic vasorelaxation in segmental arteries from both normo‐ and hypertensive rats. The effect of XE‐991, however, was not different between rat strains.

#### 3.1.2 Iso + TEA

**FIGURE 2:**
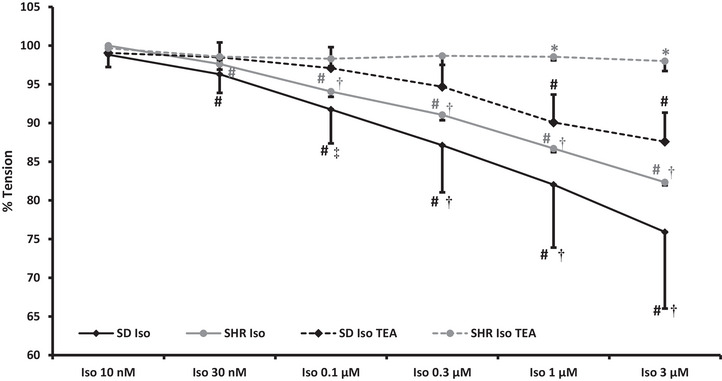
Isoprenaline elicited a significant dose‐dependent vasorelaxation (minimum tension in SD rats: 76 ± 10% and SHRs: 82 ± 1%) in isolated segmental arteries from normotensive (*n* = 6; black lines) and hypertensive (*n* = 3; grey lines) rats pre‐contracted with 3 µM methoxamine. Inhibition of BK_Ca_ channels using 1 mM TEA reduced the β‐adrenergic renal vasorelaxation significantly in both rat strains (dashed lines; tension in SD rats: 88 ± 4%) with a more pronounced effect in hypertensive rats (grey dashed line; tension remained at 98 ± 1%). A 2‐way ANOVA with repeated measures was used to compare data within strains. One‐way ANOVA was used to compared Iso 1 µM and Iso 3 µM between strains. #*P *< 0.01 versus methoxamine; ‡*P *< 0.05 versus XE; †*P *< 0.01 versus XE; *P < 0.01 versus SD. BK_Ca_, large conductance K^+^ channels; Iso, isoprenaline; SD, Sprague–Dawley; SHR, spontaneously hypertensive rat; TEA, tetraethylammonium.

Segmental vessels from six SD rats (24 vessels; ID: 344 ± 50 µm) and three SHRs (12 vessels; ID: 352 ± 59 µm; *P* = 0.696) were used to test if the β‐adrenoceptor‐induced renal vasorelaxation was reduced by closure of BK_Ca_ channels. The methoxamine‐induced vasocontraction in vessels from SD rats and SHRs before (15.0 ± 9.4 mN/2 mm vs. 18.9 ± 1.8 mN/2 mm, *P* = 0.520) or after (15.5 ± 9.7 mN/2 mm vs. 20.4 ± 1.9 mN/2 mm, *P* = 0.427) addition of TEA was not different between strains. Pre‐treatment with TEA did not significantly change baseline tension (SD rats: 0.1 ± 0.2 mN/2 mm; SHRs 0.1 ± 0.1 mN/2 mm, *P* = 0.748). However, in segmental vessels from SHRs, TEA significantly increased the methoxamine‐induced vasocontraction compared to control conditions (18.9 ± 1.8 mN/2 mm vs. 20.4 ± 1.9 mN/2 mm; *P* = 0.011). The β‐adrenergic vasorelaxation induced by isoprenaline was significant in both strains (Figure 2). The inhibition of BK_Ca_ using TEA significantly reduced the β‐adrenergic vasorelaxation in segmental vessels from normotensive rats. In segmental vessels from hypertensive rats, TEA abolished the vasorelaxation (Figure 2), and the effect of TEA was significantly stronger in segmental vessels from SHRs.

#### 3.1.3 Forsk + XE

**FIGURE 3:**
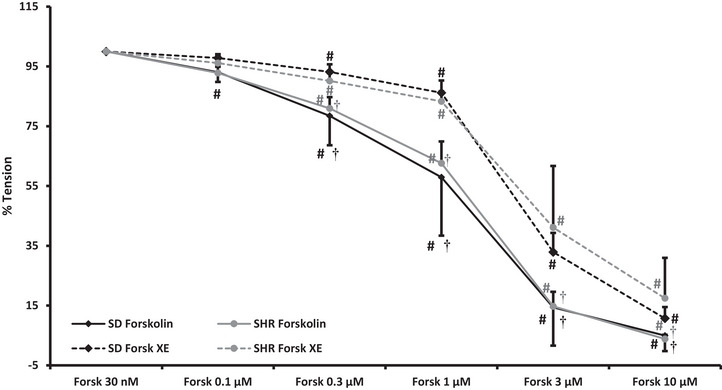
Forskolin elicited a significant dose‐dependent vasorelaxation in isolated segmental arteries from normotensive (*n* = 7; black lines; tension reduced to 5 ± 5%) and hypertensive (*n* = 6; grey lines; lowest tension 4 ± 1%) rats pre‐contracted with 3 µM methoxamine. Inhibition of K_V_7 channels using 10 M XE‐991 reduced the cAMP‐induced renal vasorelaxation significantly in both rat strains (punctured lines) in the lower concentration of forskolin. Data were compared using a 2‐way ANOVA with repeated measures. #*P *< 0.01 versus methoxamine; †*P *< 0.01 versus XE. Forsk, forskolin; SD, Sprague–Dawley; SHR, spontaneously hypertensive rat; XE, XE‐991.

Segmental vessels from seven SD rats (28 vessels; ID: 400 ± 58 µm) and six SHRs (24 vessels; ID: 336 ± 71 µm; *P* = 0.001 vs. ID from SD rats) were used for this protocol assessing if the renal vasorelaxation induced by direct activation of adenylate cyclase was reduced by closure of K_V_7 channels. Due to a smaller variation in methoxamine‐induced vasocontraction in SD rats, there was a significant difference between SD rats and SHRs before (23.1 ± 1.9 mN/2 mm vs. 19.3 ± 2.4 mN/2 mm, respectively; *P* = 0.037) and after adding XE‐991 (22.9 ± 2.2 mN/2 mm vs. 19.6 ± 2.4 mN/2 mm; *P* = 0.045). Forskolin induced a more pronounced vasorelaxation than isoprenaline in segmental vessels from both normo‐ and hypertensive rats (Figure 3). IC_50_ was not significantly different between SD rats and SHRs (0.63 ± 0.11 µM vs. 0.76 ± 0.14 µM, *P* = 0.119). Addition of XE‐991 again slightly increased baseline tension in SD rats (0.6 ± 0.3 mN/2 mm) and SHRs (0.4 ± 0.3 mN/2 mm). Closure of K_V_7 channels right shifted the vasorelaxation curves in vessels from both rat strains. IC_50_ increased significantly in SD rats to 1.4 ± 0.21 µM (*P* < 0.001 vs. control) and to 1.2 ± 0.16 µM in SHRs (*P* < 0.001 vs. control) but again there was no significant difference between strains (*P* = 0.118).

### 3.2 Isometric myograph recordings in mice

**FIGURE 4:**
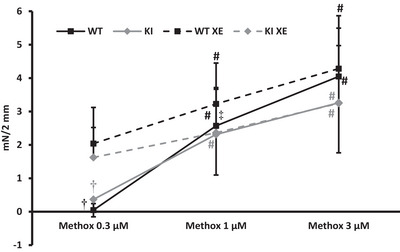
Methoxamine elicited a significant dose‐dependent vasocontraction in isolated segmental arteries from wild‐type mice (*n* = 9; black lines; max contraction: 4 ± 1 mN/2 mm) and mice lacking a functional K_V_7.1 channel (*n* = 7; grey lines; max contraction 3 ± 2 mN/2 mm). Inhibition of K_V_7 channels using 10 µM XE‐991 increased tension at the lowest methoxamine dose in both mouse strains (punctured lines; WT 2 ± 1 mN/2 mm and KI 2 ± 1 mN/2 mm) but did not significantly alter the maximum vasocontraction (WT 4 ± 2 mN/2 mm and KI 3 ± 2 mN/2 mm). A 2‐way ANOVA with repeated measures was used to compare data. #*P *< 0.01 versus 0.3 µM methoxamine; ‡*P *< 0.05 versus XE; †*P *< 0.01 versus XE. KI, knock‐in; Methox, methoxamine; mN, millinewton; WT, wild‐type; XE, XE‐991.

A total of 16 vessels from nine WT mice (seven males and two females; ID: 226 ± 46 µm) and 13 vessels from seven K_V_7.1 KI mice (four males and three females; ID: 213 ± 34 µm; NS vs. WT; *P* = 0.204) were used. The contraction elicited by KPSS and 10 µM NA was not different between WT and KI (4.4 ± 1.8 mN/2 mm vs. 3.1 ± 1.6 mN/2 mm, *P* = 0.173). Methoxamine added in cumulative doses (0.3, 1, and 3 µM) elicited a significant vasocontraction in segmental vessels from both WT and KI mice (Figure 4). When XE‐991 was added to the myograph chambers, a baseline contraction was seen (WT: 0.9 ± 0.5 mN/2 mm; KI: 0.9 ± 0.3 mN/2 mm) and the response to 0.3 µM methoxamine was significantly increased compared to the control situation. Increasing doses of methoxamine again elicited an increasing vasocontraction and the response to 3 µM methoxamine after inhibition of K_V_7 channels was not different within or between WT and KI (Figure 4).

**FIGURE 5:**
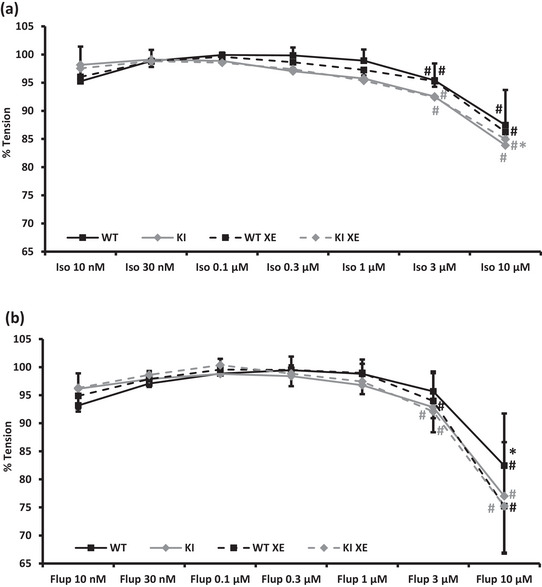
(a) In isolated pre‐contracted segmental arteries from wild‐type mice (*n* = 9; black lines) and mice lacking a functional K_V_7.1 channel (*n* = 7; grey lines), isoprenaline elicited a significant dose‐dependent vasorelaxation (minimum tension WT: 87 ± 6%; KI: 84 ± 9%), which was largely unaffected by inhibition of K_V_7 channels using 10 µM XE‐991 (dashed lines, min. tension WT: 86 ± 8%; KI: 85 ± 5%). (b) The K_V_7.2–7.5 opener flupirtine also induced a significant dose‐dependent vasorelaxation (min. tension WT: 82 ± 9%; KI: 77 ± 10%) that was not reduced by inhibition of K_V_7 channels (min. tension WT: 75 ± 11%; KI: 75 ± 8%). Data were compared using a 2‐way ANOVA with repeated measures. #*P *< 0.01 versus methoxamine; *P < 0.05 versus XE. Flup, flupirtine; Iso, isoprenaline; KI, knock‐in; WT, wild‐type; XE, XE‐991.

Addition of increasing doses of isoprenaline (10 nM to 10 µM) elicited a significant vasorelaxation in the highest doses in segmental vessels from both WT and KI mice (Figure 5a). This vasorelaxation was only changed significantly after inhibition of K_V_7 channels by XE‐991 in segmental vessels from Kv7.1 KI at the highest dose of isoprenaline (*P* = 0.011). To further assess the role of K_V_7.1 channels in the renal vasculature, the K_V_7.2–7.5 opener flupirtine was added in increasing doses (10 nM to 10 µM; Figure 5b). This also induced a significant vasorelaxation in the highest concentrations in WT and KI vessels. Interestingly, this vasorelaxation was not reduced by the K_V_7 channel inhibitor, XE‐991 (Figure 5b).

### 3.3 in vivo measurement of rat renal blood flow

The responses obtained in vitro in vessels from normo‐ and hypertensive rat, were further investigated in vivo in normotensive rats. A total of 17 SD rats weighing 314 ± 5 g were used in three different protocols.

#### 3.3.1 Iso + XE

**FIGURE 6:**
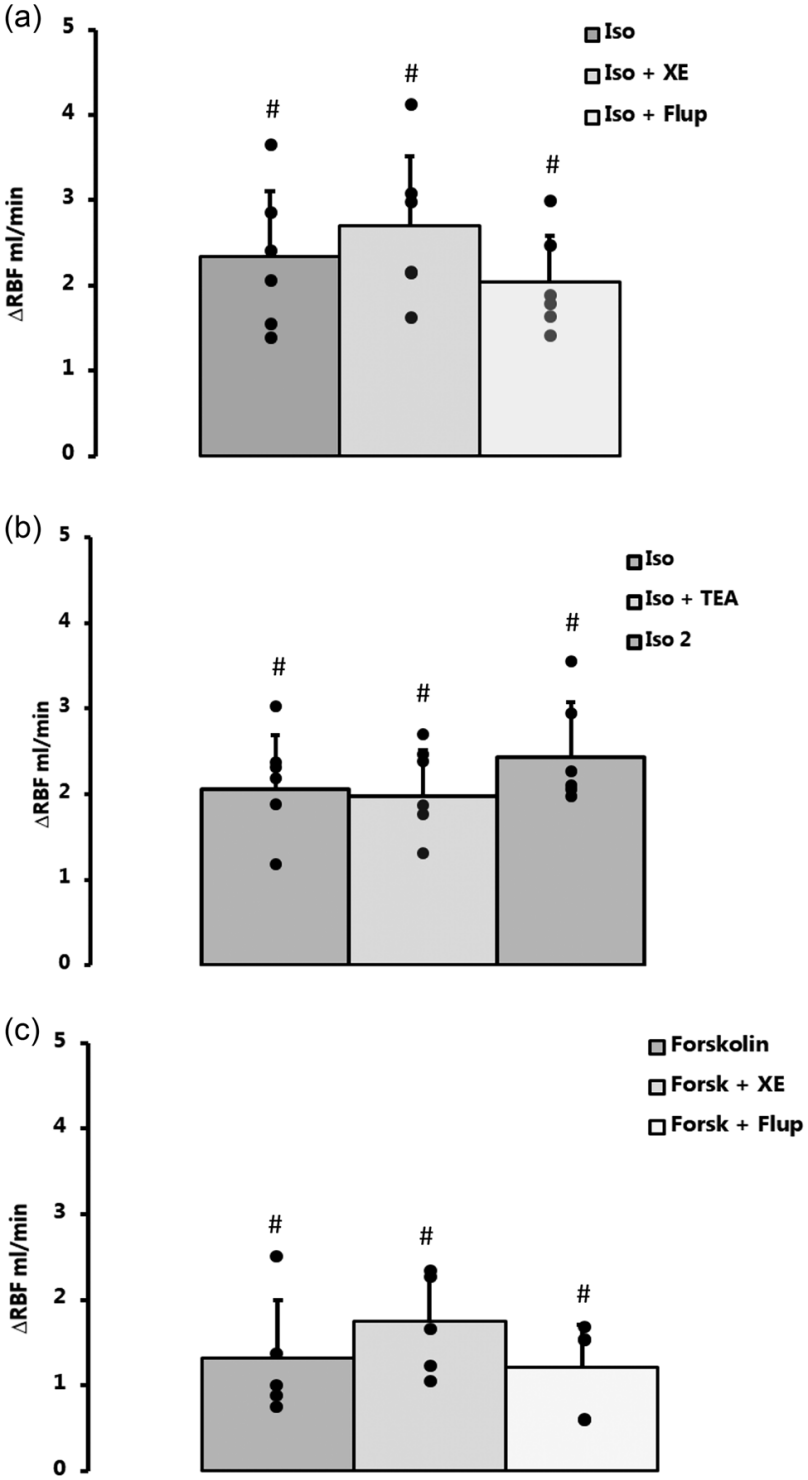
Intrarenal infusion of 0.1 µM isoprenaline elicited a significant vasodilatation in anaesthetized rats. (a) Inhibition of K_V_7 channels by 4 min pretreatment with 5 µM XE‐991 significantly decreased baseline RBF, but did not reduce the isoprenaline‐induced vasodilatation (Iso + XE) and neither did opening of K_V_7.2‐7.5 channels using 10 µM flupirtine (Iso + Flup; *n* = 6 indicated by black dots). (b) Inhibition of BK_Ca_ channels using 4 min pretreatment with 1 mM TEA also decreased baseline RBF, but did not affect the isoprenaline‐induced renal vasodilatation (Iso + TEA; *n* = 5 indicated by black dots). (c) Activation of adenylate cyclase induced a significant renal vasodilatation (Forskolin) that was not affected by inhibition (Forsk + XE) or activation (Forsk + Flup) of K_V_7 channels (*n* = 5 indicated by black dots). Data were compared using a 1‐way ANOVA with repeated measures. #*P *< 0.01 versus baseline RBF. Flup, flupirtine; Forsk, forskolin; Iso, isoprenaline; RBF, renal blood flow; TEA, tetraethylammonium; XE, XE‐991.

In six rats (mean arterial pressure (MAP) 105 ± 5 mmHg and RBF 9.2 ± 1.6 mL/min) intrarenal infusion of 0.1 µM isoprenaline elicited a significant increase in RBF (11.5 ± 2.2 mL/min, *P *< 0.001; ∆RBF shown in Figure 6a). Pre‐treatment with XE‐991 (5 µM) did not significantly affect MAP (104 ± 5 mmHg) but induced a small but significant reduction in baseline RBF (from 9.4 ± 1.4 mL/min to 9.0 ± 1.4 mm/min; *P* = 0.001). The RBF response to isoprenaline infusion was, however, not altered (11.8 ± 2.0 mL/min, *P* = 0.638; ∆RBF in Figure 6a). Pre‐treatment with flupirtine (10 µM) did not affect MAP (104 ± 4 mmHg) whereas baseline RBF increased significantly (10.0 ± 2.3 mL/min; *P* = 0.042). Intrarenal infusion of isoprenaline further increased RBF significantly, but the RBF response was not significantly different from isoprenaline alone (12.1 ± 2.6 mL/min, *P* = 0.569; ∆RBF in Figure 6a). There was no significant effect of closing or opening K_V_7 channels on the isoprenaline‐induced renal vasodilatation in vivo.

#### 3.3.2 Iso + TEA

In these six rats, MAP averaged 98 ± 8 mmHg and RBF 9.6 ± 2.1 mL/min. Again, intrarenal infusion of 0.1 µM isoprenaline induced a significant increase in RBF (11.6 ± 2.5 mL/min, *P* = 0.002; ∆RBF in Figure 6b). Pre‐treatment with 1 mM TEA slightly decreased MAP (101 ± 9 mmHg vs. 93 ± 8 mmHg; *P* = 0.003) and baseline RBF (8.0 ± 1.4 mL/min vs. 7.5 ± 1.4; *P* = 0.024). Co‐administration of TEA and isoprenaline increased RBF significantly (10.3 ± 2.0 mL/min, *P* = 0.006; ∆RBF in Figure 6b), but there was no difference compared to the increase in RBF seen with isoprenaline alone.

#### 3.3.3 Forsk + XE

In five rats with MAP 103 ± 8 mmHg and RBF 8.6 ± 2.1 mL/min, intrarenal infusion of 0.5 µM forskolin elicited a significant increase in RBF (9.9 ± 2.4 mL/min, *P* = 0.009; ∆RBF in Figure 6c). Pre‐treatment with 5 µM XE‐991 induced a slight but significant increase in MAP (101 ± 10 mmHg vs. 104 ± 9 mmHg; *P* = 0.019) and a decrease in baseline RBF (9.1 ± 2.3 mL/min vs. 8.6 ± 2.2 mL/min; *P* = 0.018). Co‐infusion of XE‐991 and forskolin increased RBF significantly (10.4 ± 2.7 mL/min, *P* = 0.001; ∆RBF in Figure 6c), but the RBF response was not different from the response elicited by forskolin alone (*P* = 0.379). Hereafter, the rats were pre‐treated with 10 µM flupirtine, which increased MAP from 102 ± 10 mmHg to 104 ± 10 mmHg (*P* = 0.035). Concurrent intrarenal infusion of 0.5 µM forskolin again increased RBF significantly (9.3 ± 2.5 mL/min vs. 10.5 ± 2.7 mL/min, *P *< 0.001; ∆RBF in Figure 6c) but the RBF response was similar to the response elicited by forskolin alone (*P* = 0.316). There was no significant effect of closing or opening K_V_7 channels on the forskolin‐induced renal vasodilatation in vivo.

## 4 DISCUSSION

The overall aim of this study was to elucidate the role of K_V_7 and BK_Ca_ channels in the β‐adrenergic vasodilatation in the renal vasculature in vivo and in vitro. We hypothesized that opening of K_V_7 channels would play a significant role in the renal β‐adrenergic relaxation in vivo and in vitro in normo‐ and hypertensive rats. We further hypothesized that K_V_7.1 would play a significant role in the β‐adrenergic renal vasorelaxation in vitro in mice. Our study shows that in isolated segmental arteries from normo‐ and hypertensive rats, β‐adrenergic vasorelaxation was equal in both strains, and it was significantly reduced by closure of K_V_7 channels and BK_Ca_ channels. The β‐adrenergic vasorelaxation was similar in segmental vessels from WT mice and mice lacking functional K_V_7.1 channels. Only at the highest dose of isoprenaline did closure of K_V_7 channels elicit a smaller vasorelaxation in segmental arteries from the KI mice. in vivo we showed that the significant increase in renal blood flow elicited by isoprenaline infusion was not affected by closure of K_V_7 channels using XE‐991 or BK_Ca_ channels using TEA, in spite of both substances inducing a significant decrease in baseline RBF in anaesthetized rats.

Stimulation of the β‐adrenergic receptors increases the intracellular levels of cAMP leading to activation of protein kinase A and opening of vascular K^+^ channels enabling a vasodilatation. The renal vasculature expresses a number of different K^+^ channels, including voltage‐sensitive K_V_ channels and calcium‐sensitive BK_Ca_ channels (Salomonsson et al., 2017). Members of both the K_V_1 and the K_V_7 family have been found in the renal vasculature, but in contrast to the K_V_1 family, the K_V_7 channels are active at more negative membrane potentials (−60 to −40 mV) (Mackie & Byron, 2008). This range is within the resting membrane potential of renal vascular smooth muscle cells (Loutzenhiser et al., 1997) suggesting that K_V_7 channels in general play a role in maintaining renal vascular resistance at baseline. Closure of K_V_7 channels in isolated segmental arteries using the non‐specific K_V_7 channel inhibitor XE‐991, elicited a dose‐dependent increase in tension (Salomonsson et al., 2015), which was abolished by the L‐type Ca^2+^ channel inhibitor nifedipine. This suggests that closure of K_V_7 channels depolarizes the renal vascular smooth muscle cells, leading to opening of the L‐type Ca^2+^ channels and vasocontraction. Furthermore, in anaesthetized rats, intra‐renal infusion of the K_V_7.2–7.5 opener flupirtine (10 µM) elicited a significant increase in renal blood flow, while XE‐991 decreased RBF, suggesting that the K_V_7 channels are active during baseline conditions in vivo (Salomonsson et al., 2015). Expression of K_V_7.1, 7.4 and 7.5 channels in vascular smooth muscle cells from the rat renal artery has been demonstrated (Chadha, Zunke, Zhu et al., 2012). However, another study failed to verify K_V_7.1 and 7.5 expression in afferent arterioles (Salomonsson et al., 2015), possibly reflecting the use of non‐specific antibodies or that the expression pattern of K_V_7 channels may vary with vessel size as previously shown for K_ir_ channels (Chilton et al., 2011), where expression decreased with increasing vessel diameter.

In the present study, increasing doses of isoprenaline elicited a significant vasorelaxation that was only partly inhibited by closure of K_V_7 channels, indicating that other mechanisms or other K^+^ channels are also involved in the β‐adrenoceptor‐induced vasodilatation of segmental arteries. This was found in vessels from both normo‐ and hypertensive rats suggesting that there is no difference in the K_V_7 channel response between normo‐ and hypertensive rats after activation of β‐adrenergic receptors in vitro. This is in contrast to data from Chada et al. who showed that the β‐adrenergic vasodilatation was not present in renal arteries from hypertensive rats (Chadha, Zunke, Zhu et al., 2012). It is possible that renal arteries of different sizes exhibit differences in vascular remodelling in the SHRs (Humphrey, 2021). Renal vascular resistance is increased in young SHRs (Dilley et al., 1984) suggesting a general renal vasoconstriction in smaller resistance arteries. However, renal vasorelaxing responses in segmental arteries from normotensive and hypertensive rats were similar in the present study. Also, in vivo studies have shown significant renal vasodilatation in SHRs during infusion of isoprenaline (Abdulla et al., 2008). Possibly, smaller renal arteries in the hypertensive kidney do possess the ability to dilate in response to β‐adrenergic receptor activation. Another difference between the present data and Chadha et al. is the smaller vasorelaxation elicited in segmental arteries from normotensive rats in our hands. One study has shown that isoprenaline‐induced vasodilatation in mesenteric arteries from SHRs is partly dependent on the size of the pre‐contraction (Blankesteijn et al., 1996). This may explain why others (Chadha, Zunke, Zhu et al., 2012; Stott et al., 2016) showed a more pronounced vasodilatory effect of isoprenaline in the renal artery, as the tension elicited by 3 µM methoxamine in those experiments was lower than in the present and other studies (Lindman et al., 2018). Also, it has previously been shown that the renal vascular bed is less sensitive to β‐adrenergic vasodilatation compared to the mesenteric vascular bed (Heesen & De Mey, 1990). However, the difference in vasorelaxing responses between studies could also be caused by the use of differently sized arteries (renal artery vs. segmental arteries).

BK_Ca_ channels are activated by PKA (Minami et al., 1993) and may therefore also be activated during β‐adrenergic stimulation as previously shown in mesenteric (Huang et al., 1998) and femoral arteries (Fujimoto et al., 2001). In coronary arteries, BK_Ca_ channels are activated during stimulation with both isoprenaline and forskolin (White et al., 2000). In mesenteric arteries, isoprenaline‐induced vasodilatation was significantly reduced by TEA (White et al., 2001) or iberiotoxin, a more specific inhibitor of BK_Ca_ channels (Matsumoto et al., 2012). We therefore also tested the effect of TEA on β‐adrenergic renal vasodilatation. Previously, TEA (but not iberiotoxin) has been shown to elicit a significant renal vasoconstriction in vivo in normotensive rats (Magnusson et al., 2007; Sorensen et al., 2011). At the chosen concentration, TEA is believed to primarily inhibit BK_Ca_ channels (Orito et al., 1994; Su et al., 2001). In isolated segmental arteries from normo‐ and hypertensive rats, TEA significantly reduced the β‐adrenergic vasodilatation, suggesting that BK_Ca_ channels also play a significant role in this regard. Interestingly, the inhibition of BK_Ca_ channels had a more pronounced effect on the β‐receptor‐induced renal vasorelaxation in segmental arteries from hypertensive rats. In hypertensive DOCA‐salt rats it was found that the activity of BK_Ca_ channels in mesenteric arteries was reduced compared to normotensive rats and this partly caused a decreased vasodilatory effect of isoprenaline (Matsumoto et al., 2012). In SHRs, a decreased expression of the BK_Ca_ channel β1 subunit was found in cerebral arteries, which caused a decrease in Ca^2+^ sensitivity (Amberg & Santana, 2003). In the renal segmental arteries used in this study, BK_Ca_ channels seem to play a significant role in the β‐adrenergic vasodilatation and even more so in vessels from hypertensive rats.

To further stimulate the renal vasodilatation, we used forskolin to increase the level of cAMP. This induced a more prominent vasodilatation compared to isoprenaline in vessels from both normo‐ and hypertensive rats. Using renal segmental arteries from stroke‐prone hypertensive rats and normotensive Wistar Kyoto rats, it was previously shown that the relaxing effect of forskolin is similar between strains (Gao et al., 1994). Interestingly, at lower doses of forskolin, inhibition of K_V_7 channels effectively reduced the vasodilatory effect, but at higher concentrations of forskolin, giving a more pronounced vasodilatation, the effect of XE‐991 was less prominent. This suggests that higher concentrations of cAMP and thus increased activation of PKA is activating other classes of K^+^ channels, for example, BK_Ca_ channels. in vivo we have shown that inhibition of a single class of K^+^ channels elicits only a small reduction in renal blood flow, but when several inhibitors are combined, a more prominent reduction in RBF is obtained (Sorensen et al., 2011). This finding supports the notion of different classes of K^+^ channels substituting for each other and being activated at the same time.

Expression of *KCNQ4* and K_V_7.4 has been shown to be reduced in aorta and mesenteric arteries from hypertensive rats (Jepps et al., 2011). Conversely, *KCNQ1* was found to be significantly overexpressed in mesenteric arteries from genetically hypertensive rats (SHR/SPSHR) and the 2‐kidney‐1‐clip hypertensive rat (Ikawa et al., 2019). Also, a genetic variation reducing expression of *KCNQ1* in humans correlated with a decreased risk of hypertension, and inhibition of K_V_7.1 in VSMCs significantly decreased contraction (Huang et al., 2017). Paradoxically, this suggests that lower expression or function of K_V_7.1 channels favours vasodilatation and/or opposes vasocontraction. However, activation of K_V_7.1 channels induced vasorelaxation in mesenteric arteries pre‐constricted with methoxamine (Chadha, Zunke, Davis et al., 2012) and inhibition of K_V_7.1 channels significantly reduced renal autoregulation in neonatal pigs in response to blood pressure decreases (Peixoto‐Neves et al., 2022). We therefore investigated whether a loss‐of‐function mutation in *KCNQ1* would have any effect on the methoxamine‐induced vasoconstriction or the isoprenaline‐induced vasodilatation in segmental arteries from mice. At the highest methoxamine concentration, there was a tendency towards a smaller vasocontraction in the KI mice compared to WT, which would corroborate the hypothesis that lower expression of K_V_7.1 opposes vasocontraction. However, the difference in vasocontraction was not significant. The isoprenaline‐induced vasorelaxation was similar between vessels from WT and KI mice, suggesting no significant role for K_V_7.1 in the renal β‐adrenergic vasodilatation. Interestingly, XE‐991 induced a baseline contraction in segmental vessels from both WT and KI mice, but had no effect on the isoprenaline‐induced vasorelaxation, suggesting that opening of K_V_7 channels is not involved in the β‐adrenergic renal vasodilatation in mice. Other results obtained in mice showed that XE‐991 did not inhibit the vasorelaxation elicited by a K_V_7.1 opener (R‐L3) (Tsvetkov et al., 2017). We further tested the vasorelaxing effect of the opener of K_V_7.2–7.5 channels flupirtine in murine segmental arteries. Flupirtine induced a significant vasorelaxation in vessels from both WT and KI mice, and again, the inhibition of K_V_7 channels using XE‐991 did not affect the vasodilatation. We have previously observed the same inability of XE‐991 to inhibit the renal vasodilatory effect of flupirtine in rats in vivo (Salomonsson et al., 2015).

We used renal segmental arteries from both male and female mice in the present study. It has previously been shown that the response to inhibition of K_V_7.1 channels in mesenteric arteries is different in vessels from male rats compared to vessels from female rats, although this was dependent on the oestrous cycle (Baldwin et al., 2022). In this study, only a few vessels from female mice were included, and we had no information where in the oestrous cycle the mice were at the time of the experiment. This makes it difficult to conclude on possible sex differences in the renal vascular responses here. However, previously, bolus injections of Ang II or phenylephrine elicited similar decreases in renal blood flow in male and female mice (Schneider et al., 2010). Also, no sex differences were found in glomerular filtration rate or renal blood flow in C57Bl6 mice (Tao et al., 2023) suggesting that, in general, renal vascular responses are not different between male and female mice.

In anaesthetized normotensive rats, intrarenal infusion of isoprenaline elicited a significant increase in renal blood flow, showing a renal vasodilatation. As the pre‐treatment with both the K_V_7 inhibitor and the K_V_7.2–5 opener had significant effects on baseline renal blood flow, the chosen concentrations of XE‐991 and flupirtine seem sufficient in inhibiting/activating K_V_7 channels in vivo as also shown previously (Salomonsson et al., 2015). However, none of the compounds affected the β‐adrenergic renal vasodilatation. Other in vivo results previously showed that the renal vasodilatation elicited by ACh was not affected by either flupirtine or XE‐991 (Salomonsson et al., 2015). Activation of the vascular muscarinic receptors leads to activation of protein kinase G (PKG), which is also known to activate several classes of vascular K^+^ channels (Salomonsson et al., 2017). Possibly, these findings reflect that in vivo, several classes of K^+^ channels are activated simultaneously following stimulation of β‐adrenergic receptors. We previously showed that several classes of K^+^ channels need to be activated or inhibited in vivo in order to obtain a significant response (Sorensen et al., 2011). If just one type of K^+^ channel is inhibited, only minor effects are seen on renal blood flow whereas a cocktail of several inhibitors targeting K_V_1, BK_Ca_, K_ir_, SK_Ca_ and K_ATP_ elicited a significant reduction in renal blood flow. These data underline the important differences between results obtained in vitro, where inhibition of K_ATP_ (Lorenz et al., 1992), SK_Ca_ (Gebremedhin et al., 1996; Wang & Loutzenhiser, 2002) or K_V_1 (Troncoso Brindeiro et al., 2008; Zou et al., 1996) induced significant afferent arteriolar vasoconstriction but in vivo no significant effect was seen on renal blood flow (Sorensen et al., 2011). It is possible, that the exposure of VSMC to the injected inhibitors is more limited in vivo as they are delivered intra‐vascularly, whereas in the myograph‐bath, the segmental artery is surrounded by buffer containing the inhibitor. Thus, we cannot exclude that the effect of the inhibitor is reduced in the in vivo experiment or that counteracting mechanisms are activated in the intact animal reducing the effect of the K^+^ channel inhibitors. However, as mentioned above, all the inhibitors influenced baseline blood flow, suggesting that they reached the VSMC also in vivo. Another interesting finding is that in vitro forskolin induced a stronger vasorelaxation compared to isoprenaline. However, in vivo the vasodilatory effect of isoprenaline was more pronounced compared to the effect of forskolin.

In conclusion, both K_V_7 and BK_Ca_ channels seem to participate in the renal β‐adrenergic vasodilatation in isolated renal segmental arteries from rats, but not in murine segmental arteries. In anaesthetized normotensive rats, no effect of K_V_7 and BK_Ca_ channel inhibitors was found, possibly reflecting that several other mechanisms participate in the regulation of renal blood flow in vivo. This underlines the relevance of verifying in vitro data in intact animals, where the physiological regulatory mechanisms integrate their responses to maintain renal blood flow.

## AUTHOR CONTRIBUTIONS

All experiments were performed in the laboratory of Max Salomonsson and Charlotte Mehlin Sorensen at University of Copenhagen, Health & Medical Sciences, Denmark. Charlotte M. Sorensen: Conception and design of the work; Acquisition, analysis, and interpretation of data; Drafting of the work and revising it critically for important intellectual content. Max Salomonsson: Conception and design of the work; analysis, and interpretation of data; revising the work critically for important intellectual content Anniek F. Lubberding: interpretation of data; revising the work critically for important intellectual content. Niels‐Henrik Holstein‐Rathlou: interpretation of data; revising the work critically for important intellectual content. All authors have read and approved the final version of this manuscript and agree to be accountable for all aspects of the work in ensuring that questions related to the accuracy or integrity of any part of the work are appropriately investigated and resolved. All persons designated as authors qualify for authorship, and all those who qualify for authorship are listed.

## CONFLICT OF INTEREST

None.

## FUNDING INFORMATION

None.

## Data Availability

The data supporting the findings presented in this paper are available at FigShare https://figshare.com/account/home#/projects/197836.
